# Supplementary material for: Mechanical Properties and Weibull Scaling Laws of Unknown Spider Silks
Source: Molecules. 2020 Jun 26;25(12):2938. doi: 10.3390/molecules25122938 (PMC7355793; doi:10.3390/molecules25122938)
Supplement: Supplementary file 1 [file molecules-25-02938-s001.pdf]

molecules

MDPI

Supplementary information

Mechanical Properties and Weibull Scaling Laws of Unknown Spider Silks

Gabriele Greco [1]; and Nicola M. Pugno [1, 2*]

[1] Laboratory of Bio-inspired, Bionic, Nano, Meta Materials & Mechanics,
Department of Civil, Environmental and Mechanical Engineering, University of Trento,
Via Mesiano, 77, 38123 Trento, Italy
[2] Queen Mary University of London, London, United Kingdom, Mile End Rd, London E1 4NS, United Kingdom
*Corresponding author: nicola.pugno@unitn.it

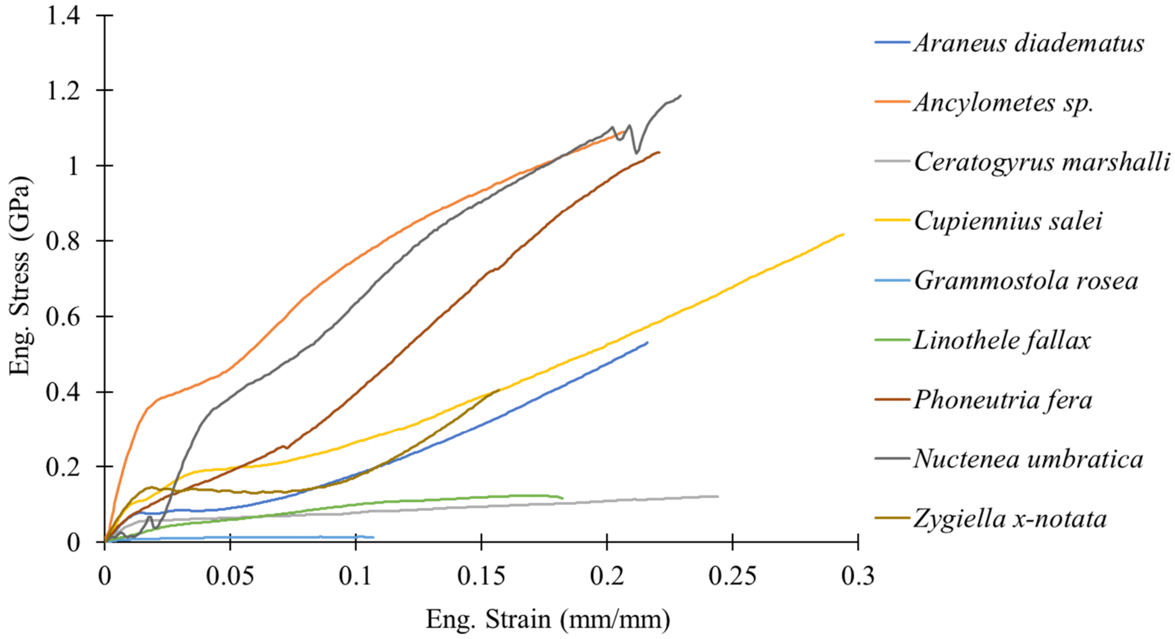

Figure S1. Representative stress-strain curves of the analyzed different types of spider silk.

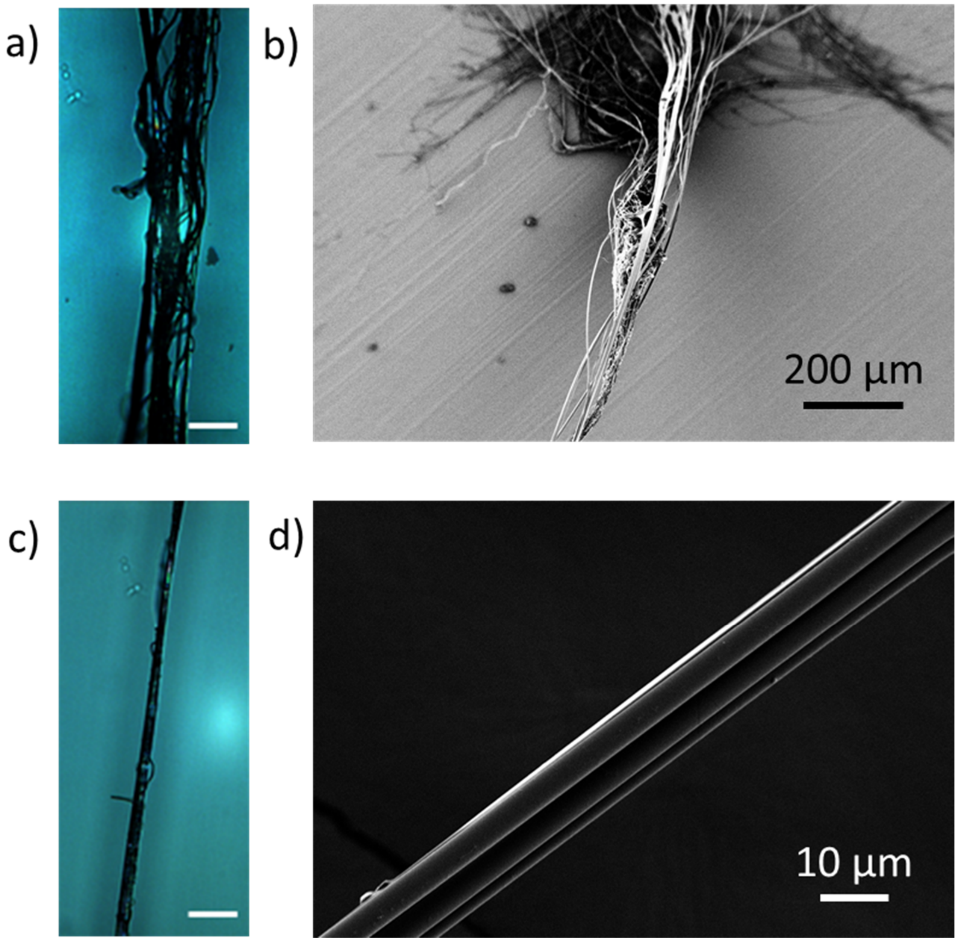

Figure S2. (a) Optical image of the dragline near the attachment discs and (b) the equivalent SEM image. (c) Optical image of the dragline at ca 1 cm from the attachment discs and the equivalent SEM image. White scale bar 50 µm. Adapted from[48].

Table S1. P-values and Cohen's d_c coefficient (in brackets) of the pairwise comparison for the strain at break at different strain rates. Only the significative differences are depicted.

Strain at break (mm/mm) p-values (Cohen's d_c)					
**Strain rate (mm/s)**	**0.08**	**0.10**	**0.11**	**0.15**	**0.17**
**0.08**			0.0376 (0.51)		
**0.10**			0.01256 (0.58)		
**0.11**	0.0376 (0.51)	0.01256 (0.58)			0.0032 (0.73)
**0.15**					
**0.17**			0.0032 (0.73)		

Table S2. P-values and Cohen's d_c coefficient (in brackets) of the pairwise comparison for the strength at different strain rates. Only the significative differences are depicted.

Strength (MPa) p-values (Cohen's d_c)					
**Strain rate (mm/s)**	**0.08**	**0.10**	**0.11**	**0.15**	**0.17**
**0.08**				0.0022 (0.80)	
**0.10**				0.001 (0.84)	
**0.11**				0.0002 (0.97)	

**0.15**	0.0022 (0.80)	0.001 (0.84)	0.0002 (0.97)		0.0001 (1.02)
**0.17**				0.0001 (1.02)	

Table S3. P-values and Cohen's d_c coefficient (in brackets) of the pairwise comparison for the Young's modulus at different strain rates. Only the significative differences are depicted.

**Young's modulus (GPa) p-values (Cohen's d_c)**					
**Strain rate (mm/s)**	**0.08**	**0.10**	**0.11**	**0.15**	**0.17**
**0.08**		0.0007 (0.41)	$5 * 10^{-6}$ (0.90)	$6 * 10^{-6}$ (1.24)	
**0.10**	0.0007 (0.41)		0.0056 (1.05)	$3 * 10^{-11}$ (0.71)	$7 * 10^{-6}$ (0.03)
**0.11**	$5 * 10^{-6}$ (0.90)	0.0056 (1.05)		$1 * 10^{-15}$ (1.93)	$1 * 10^{-7}$ (1.19)
**0.15**	$6 * 10^{-6}$ (1.24)	$3 * 10^{-11}$ (0.71)	$1 * 10^{-15}$ (1.93)		0.0022 (0.79)
**0.17**		$7 * 10^{-6}$ (0.03)	$1 * 10^{-7}$ (1.19)	0.0022 (0.79)	

Table S4. P-values and Cohen's d_c coefficient (in brackets) of the pairwise comparison for the toughness modulus at different strain rates. Only the significative differences are depicted.

**Toughness modulus (MJ/m^3) p-values (Cohen's d_c)**					
**Strain rate (mm/s)**	**0.08**	**0.10**	**0.11**	**0.15**	**0.17**
**0.08**				0.0039 (0.75)	
**0.10**				0.0237 (0.56)	
**0.11**				0.0021 (0.78)	
**0.15**	0.0039 (0.75)	0.0237 (0.56)	0.0021 (0.78)		0.0026 (0.77)
**0.17**				0.0026 (0.77)	

Table S5. P-values and Cohen's d_c coefficient (in brackets) of the pairwise comparison for the diameter at different length. Only the significative differences are depicted.

**Diameter (μm) p-values (Cohen's d_c)**					
**Length (cm)**	**0.55**	**0.75**	**1.0**	**1.25**	**1.5**
**0.55**		0.0118 (0.62)	$3 * 10^{-5}$ (1.07)	0.0006 (0.91)	0.0003 (0.93)
**0.75**	0.0118 (0.62)		0.0056 (0.67)	0.0545 (0.47)	0.0556 (0.45)
**1.0**	$3 * 10^{-5}$ (1.07)	0.0056 (0.67)			
**1.25**	0.0006 (0.91)	0.0545 (0.47)			
**1.5**	0.0003 (0.93)	0.0556 (0.45)			

Table S6. P-values and Cohen's d_c coefficient (in brackets) of the pairwise comparison for the Strength at different length. Only the significative differences are depicted.

**Strength (MPa) p-values (Cohen's d_c)**					
**Length (cm)**	**0.55**	**0.75**	**1.0**	**1.25**	**1.5**
**0.55**			0.0497 (0.54)		$1 * 10^{-6}$ (1.38)

**0.75**					0.0007 (0.92)
**1.0**	0.0497 (0.54)				0.002 (0.84)
**1.25**					0.0004 (0.96)
**1.5**	$1 * 10^{-6}$ (1.38)	0.0007 (0.92)	0.002 (0.84)	0.0004 (0.96)	
